# Book Review

**Published:** 1908-09

**Authors:** 


**Department of Neurology, Harvard Medical School.** Vol. III. Boston, Mass., U.S.A. 1908.—This volume comprises papers on neurological subjects, contributed by members of the staffs of the Massachusetts General Hospital, Boston City Hospital, Long Island Hospital and Neurological Laboratory, to various medical journals during the year 1907. These papers, which reach a high standard of excellence, and are of considerable neurological interest, are bound together and consecutively paged. They are thus in a form easily accessible for reference.

**Injuries of Nerves, and their Treatment.** By JAMES SHERREN, F.R.C.S. Pp. 310. London: Jas. Nisbet and Co. 1908.—This is an excellent book, well printed and illustrated, clearly written, not lengthy, yet containing a large amount of information given in a thoroughly practical manner, and much of it based on the author's own original work. Mr. Sherren is well known as the author of several valuable papers on the subject. His book shows originality of thought, and is obviously the outcome of a large clinical experience. It deals first with the symptoms in general produced by complete and incomplete division of a nerve, and the description of these is followed by an account of methods of examination, differential diagnosis and treatment. Plastic operations upon nerves are then carefully dealt with. The author gives a full description of the symptoms and diagnosis of injuries of each of the main nerves, and of the nerve-plexuses. There are a number of original illustrations, consisting chiefly of diagrams of areas of loss of sensation, and photographs of deformities produced by the paralysis resulting from various nerve lesions, which will be found very useful. We confidently recommend this book as embodying the most recent work on lesions of nerves, and as an excellent and practical guide to this important class of injuries.

**Insanity: its Causes and Prevention.** By CHARLES WILLIAMS. Pp. 92. London: The Ambrose Co., Ltd. 1908.—This essay contains about one hundred pages, the first half of which is chiefly an enumeration, with commentaries, of the official causes of insanity, while the second and more important half deals with the steps to be taken to remove each particular cause. The two prevailing factors in the causation of insanity are heredity and intemperance in drink, and for the former the author lays down certain rules in regard to marriage of tainted persons. These rules contain nothing new from a medical point of view, but the author's object is to remove the ignorance of the general public, and in this spirit the whole book is written. On the subject of alcoholic intemperance the suggestions for prevention do not err on the side of leniency. They are (1) distribution wholesale of forcibly plain pamphlets; (2) intemperance to be a "just cause or impediment" for a clergyman to refuse to celebrate a marriage; (3) for persistence in drunken habits after marriage, that he or she should be made to keep sober by enforcement in all cases of the Inebriates Act. The book contains many quotations from well-known writers, and is thoroughly recommendable from a popular point of view.

**Insanity and Allied Neuroses.** By GEORGE H. SAVAGE, M.D., with the assistance of EDWIN GOODALL, M.D. New and Enlarged Edition. Pp. xiv., 624. London: Cassell & Company Limited. 1907.—This book has been thoroughly revised and brought up to date with the assistance of Dr. Goodall. Several of the articles—notably those on general paralysis of the insane, moral insanity and paranoia—have been largely rewritten, and contain much new matter. An important feature is the addition of many pathological illustrations, but the authors have wisely refrained from materially increasing the size of the volume (only to the extent of about fifty pages), so that it still remains within a handy compass. In its new form it is well adapted to the needs of the ordinary curriculum, and is also a useful practical guide for the busy practitioner.

